# Endovascular Treatment of Renal Artery Pseudoaneurysm Following Laparoscopic Partial Nephrectomy: A Case Report

**DOI:** 10.7759/cureus.76764

**Published:** 2025-01-01

**Authors:** Luis C Salazar, Agustina M Suarez Anzorena, Francisco J Suarez Anzorena

**Affiliations:** 1 Interventional Radiology, Universidad de Buenos Aires, Ciudad Autonoma de Buenos Aires, ARG

**Keywords:** angiography, embolization, false aneurysm, nephrectomy, renal cell carcinoma

## Abstract

This report presents the case of a patient diagnosed with stage I renal cell carcinoma (RCC), who was treated with a laparoscopic partial nephrectomy and, subsequently, percutaneous transarterial embolization (PTAE) due to a renal pseudoaneurysm secondary to partial nephrectomy. A laparoscopic partial nephrectomy was performed, sparing the healthy renal tissue. Four days after surgery, the patient’s hematocrit count decreased by 8 points, and macroscopic hematuria was noted. PTAE of a renal pseudoaneurysm was indicated due to refractory bleeding. Adequate bleeding control and preservation of the renal parenchyma were achieved. Progress in early diagnosis of malignancy has resulted in the detection of more than 60% of RCCs at stage T1. In this scenario, new surgical techniques, which spare the renal parenchyma, have become the recommended therapeutic option in patients with early renal tumors. Likewise, dealing with complications arising from these new surgical techniques should also focus on maintaining kidney tissue integrity. We highlight the growing trend to implement minimally invasive therapies in patient care while pointing out the scarcity of studies contrasting immediate imaging results and clinically significant outcomes.

## Introduction

Renal cell carcinoma (RCC) accounts for 3% of all malignant tumors worldwide [1]. Historically, the gold standard treatment for non-metastatic RCC has been an open surgical approach [2]; however, the first laparoscopic partial nephrectomy (LPN) was performed by urologist Howard Winfield in 1992 in the United States on a patient with a non-cancerous renal tumor [3].

Progress in early diagnosis of malignancy has resulted in the detection of more than 60% of RCCs at stage T1 [4]. In this scenario, new surgical techniques, which spare the renal parenchyma, have become the recommended therapeutic option in patients with early renal tumors, without relevant differences in oncological outcomes compared with radical nephrectomy (laparoscopic, robot-assisted, or open) [5]. One of such therapeutic options is LPN, which, in general, is associated with lower rates of complications compared to open partial nephrectomy, with the exception of renal pseudoaneurysms, for which there is no significant difference between the two techniques (RR: 0.69 [0.42; 1.13]) [2].

Renal pseudoaneurysm refers to a disruption of the arterial wall contained by one or two vessel layers or solely by soft tissue, with its lobulated form arising from the compressive influence of adjacent renal tissue, typical of pseudoaneurysms [6]. This rare condition is not only a complication of LPN, but can also result from trauma, infection, neoplasia, or iatrogenic causes. The latter includes open surgical techniques, ablation, robotic surgery, ureteroscopic lithotripsy, renal biopsies, nephrostomies, percutaneous nephrolithotomy, endovascular procedures, and even post-renal transplantation [7].

Percutaneous transarterial embolization (PTAE) is the first-line therapy for the treatment of renal pseudoaneurysms [6]. It was first performed by Lars E. Almgård in 1972 to reduce the tumor size of RCC prior to surgery [8]. PTAE of renal pseudoaneurysm can be performed using polyvinyl alcohol (PVA) particles, gelatin sponge, and microcoils, either individually or in combination.

Nephrectomy is recommended for the treatment of renal pseudoaneurysms only when interventional treatment is not feasible, PTAE fails repeatedly, recanalization occurs, and endovascular treatment or contrast medium is contraindicated. In contrast, open vascular surgery with extra-anatomic bypass or autologous graft is an option in selected cases, including mycotic pseudoaneurysm, a proximal location of the pseudoaneurysm at the level of the main renal artery, and in patients who have received renal transplantation. Additionally, immunosuppressive therapy is necessary in conjunction with endovascular treatment for renal pseudoaneurysms that are secondary to Behçet's disease [6].

Despite reports of direct percutaneous embolization for renal pseudoaneurysms using various embolization and/or thrombogenic drugs, the efficacy of this method remains poorly studied [9]. Although spontaneous resolution has been documented in certain small pseudoaneurysms, this is atypical, as the hemorrhage associated with renal pseudoaneurysms is generally progressive and potentially life-threatening [6].

The following report describes the endovascular treatment of a renal pseudoaneurysm secondary to LPN as part of the management of stage I RCC. It illustrates the current approach to minimally invasive alternatives for both early oncologic treatment and potential complications.

## Case presentation

**Figure 1:**
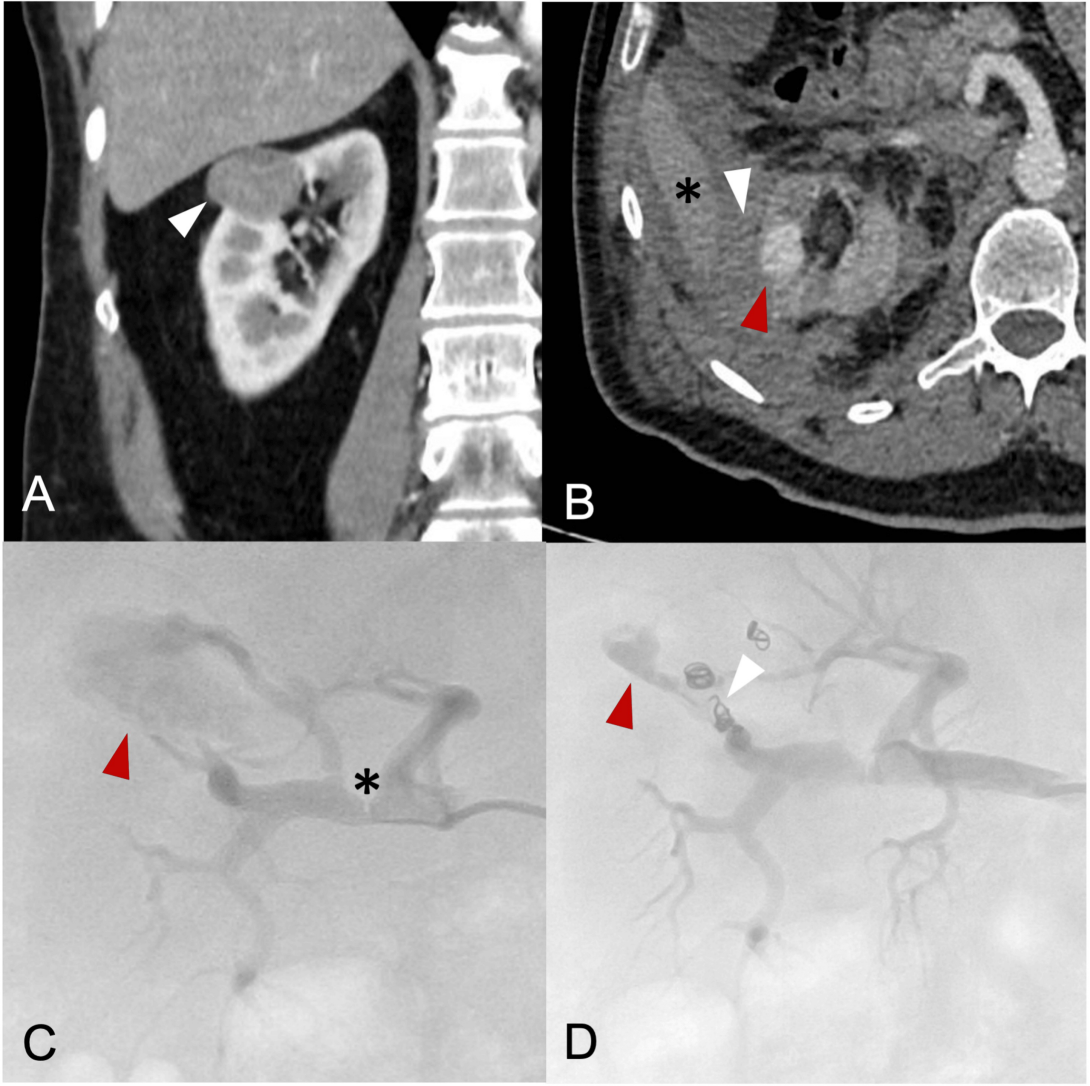
CT and Angiography A: Coronal CT, neoplastic lesion in the right kidney (arrowhead). B: Axial CT, pseudoaneurysm (red arrowhead), hemorrhage in Morrison's pouch (white arrowhead), liver (asterisk). C: Pre-embolization angiography, AP projection of right kidney, pseudoaneurysm (arrowhead), stenosis (asterisk). D: Post-embolization angiography, AP projection of right kidney, small remnant (red arrowhead), and microcoils (white arrowhead). AP, anteroposterior.

A 75-year-old male patient with a history of smoking, hypertension, dyslipidemia, hypothyroidism, and familial predisposition to renal cancer (brother underwent total nephrectomy) attended a medical consultation due to the incidental finding of a solid lesion in the interpolar region of the right kidney (RENAL Nephrometry Score of 8 points, indicating intermediate risk of surgical complications) (Figure 1A). To treat the lesion, an LPN was performed, sparing the healthy renal parenchyma. The pathological report revealed a moderately differentiated renal cell adenocarcinoma.

Four days after surgery, the patient’s hematocrit count decreased by 8 points, and macroscopic hematuria was noted, requiring the transfusion of two units of red blood cells. A CT scan showed surgical changes resulting from an LPN performed on the right side via anterior approach, in addition to renal and right perirenal hemorrhage extending to the Morrison's pouch. A CT angiography showed a saccular formation related to the surgical bed within the upper interpolar region of the right kidney, consistent with renal pseudoaneurysm (Figure 1B). Renal PTAE was indicated due to persistent hemorrhage.

The procedure was performed in an angiography room using a monoplane unit, with conscious sedation and cardiorespiratory monitoring. Before the procedure, the patient was administered prophylactic antibiotics. A 5 Fr vascular introducer facilitated the advancement of a 5 Fr cobra catheter through the right inguinal canal to the right main renal artery, identifying a 25 mm pseudoaneurysm associated with the right superior and anterior superior segmental arteries of the lobar branches, alongside atherosclerotic stenosis of the anterior segmental artery of 80%. A Progreat™ 2.7 Fr microcatheter (Terumo, Tokyo, Japan) with microguidewire was advanced using a coaxial method, resulting in superselective catheterization of the lesion. Embolization with microcoils and Spongostan™ was then performed, achieving significant flow reduction within the lesion, resulting in the vanishing of most of the pseudoaneurysm at the end of the procedure. 

Finally, hematuria subsided, hematocrit levels stabilized, and the patient was discharged two days after embolization, with a recommendation for outpatient follow-up.

## Discussion

Renal hemorrhage in a patient who has undergone nephron-sparing surgery is a potentially life-threatening complication occurring in 8.3% of cases [10]; hence, early diagnosis and timely treatment are essential components of care. The most frequent symptoms of renal hemorrhage include persistent gross hematuria, abdominal or lower back pain, and hypovolemic shock. Conservative treatment of renal pseudoaneurysm is associated with low effectiveness and high recurrence, while PTAE has shown a significant decrease in complications and better performance in renal function tests compared to radical nephrectomy [11].

Percutaneous angiography prior to embolization is capable of characterizing the vascular anatomy with greater precision compared to CT angiography. However, in cases of traumatic pseudoaneurysm, such as renal hemorrhage after surgery, the occurrence of vasospasms should be considered for imaging interpretation and treatment strategies.

The selection of the embolizing agent is based primarily on blood flow velocity, the size of the arterial vessels involved, the desired occlusion time (temporary or permanent), and the availability of resources. Both PVA particles and coils have low recanalization rates [11].

In cases of a traumatic lesion in the branches of the main renal artery with confirmed active hemorrhage on angiography, coils adapted to the size of the vessel provide a rapid and long-lasting solution. Meanwhile, in cases of small renal pseudoaneurysms secondary to trauma, it is recommended to use a combination of PVA particles and gelatin sponges for treatment. Finally, in individuals with large pseudoaneurysms, whether due to trauma or medical intervention, in addition to coils, gelatin sponges can be used to prevent coil migration [11]. However, even though particles perform well in neoplastic lesions, they have a lower performance in localized lesions (e.g., traumatic or iatrogenic), which carry a high risk of nontarget embolization due to reflux [12].

Our patient presented with a 25 mm iatrogenic pseudoaneurysm with two main arterial afferents (Figure 1C), which were embolized using four microcoils reinforced with Spongostan™ (Figure 1D). Albeit, enacting this therapeutic plan, we did not achieve the complete occlusion of the lesion with the embolic material, we attained both clinical success and the absence of new complications associated with the intervention. Clinical success was considered because of the inexistence of repetitive bleeding episodes during follow-up, without the need for a new endovascular intervention or a subsequent surgical approach [13]. This favorable outcome was likely the result of multiple factors. First, the thrombogenic capacity of the materials used [14]; second, the patient's hematologic condition; and third, the flow reduction within the pseudoaneurysm. In this case, all the afferent vessels of the pseudoaneurysm were properly identified and directly managed.

Before choosing the therapeutic strategy, it is important to assess the angioarchitecture and flow dynamics of the lesion. In our case, it was possible to achieve a satisfactory clinical outcome, while indirectly reducing material costs and operating and radiation times compared to therapeutic approaches that aim at complete and immediate angiographic occlusion of the lesion.

Some authors [15-18] have reported the need to apply a serial protocol through multiple PTAE sessions to treat renal pseudoaneurysm patients when neither technical nor clinical success is achieved in the first PTAE session. In our case, clinical success was reached with a single session.

Even though some literature has suggested the need to reduce the number of coils used in the treatment of pseudoaneurysms [19], it is still insufficient. Further research is required to evaluate intermediate outcomes in the treatment of renal pseudoaneurysms, such as immediate complete occlusion on angiography vs. incomplete embolization, while controlling for variables including the number of afferents, the morphology and dimensions of the lesion, the type of embolizing agent employed, and the time required for pseudoaneurysms to achieve complete thrombosis, among others.

The therapeutic strategy selected must not only take into account the morphology and physiology of the pseudoaneurysm, but also the thrombogenic potential of the embolizing material and the hematologic and hemodynamic characteristics of each patient. In other words, more research is needed to establish the probability of complete occlusion and/or clinical improvement in patients with incomplete embolization of renal pseudoaneurysms. It should be noted that demonstrating complete occlusion of the renal pseudoaneurysm through imaging is not synonymous with cure, given that some cases of recanalization and/or recurrence of the lesion have been reported [6,20].

## Conclusions

This is the report of a patient with an incidental diagnosis of stage I RCC, who was treated by LPN using the parenchymal sparing surgery technique. In addition, PTAE of a renal pseudoaneurysm secondary to partial nephrectomy was performed. Adequate bleeding control and patient stabilization were achieved.

This case illustrates the current search for minimally invasive alternatives with a better cost-benefit ratio, which is crucial for both early oncologic treatment and the potential complications that may arise from it.
